# Mucosal B Cell Expansion and Maturation Contribute to Colitis Pathogenesis

**DOI:** 10.1093/ibd/izaf275

**Published:** 2025-11-20

**Authors:** Ivan C N Fung, Pim J Koelink, Lieven G M Mulders, Iris Admiraal, Caroline Verseijden, Jan Verhoeff, Manon E Wildenberg, Yi Luo, Geert R D’Haens, Andrew Y F Li Yim, Wouter J de Jonge

**Affiliations:** Tytgat Institute for Liver and Intestinal Research, Amsterdam UMC, Location University of Amsterdam, Amsterdam, The Netherlands; Amsterdam Gastroenterology Endocrinology Metabolism (AGEM), Amsterdam, The Netherlands; Tytgat Institute for Liver and Intestinal Research, Amsterdam UMC, Location University of Amsterdam, Amsterdam, The Netherlands; Amsterdam Gastroenterology Endocrinology Metabolism (AGEM), Amsterdam, The Netherlands; Department of Gastroenterology and Hepatology, Amsterdam UMC, Location University of Amsterdam, Amsterdam, The Netherlands; Tytgat Institute for Liver and Intestinal Research, Amsterdam UMC, Location University of Amsterdam, Amsterdam, The Netherlands; Tytgat Institute for Liver and Intestinal Research, Amsterdam UMC, Location University of Amsterdam, Amsterdam, The Netherlands; Tytgat Institute for Liver and Intestinal Research, Amsterdam UMC, Location University of Amsterdam, Amsterdam, The Netherlands; Amsterdam Gastroenterology Endocrinology Metabolism (AGEM), Amsterdam, The Netherlands; Department of Molecular Cell Biology & Immunology, Amsterdam Infection & Immunity Institute and Cancer Center Amsterdam, Amsterdam University Medical Centers, Free University Amsterdam, Amsterdam, The Netherlands; Tytgat Institute for Liver and Intestinal Research, Amsterdam UMC, Location University of Amsterdam, Amsterdam, The Netherlands; Amsterdam Gastroenterology Endocrinology Metabolism (AGEM), Amsterdam, The Netherlands; Department of Gastroenterology and Hepatology, Amsterdam UMC, Location University of Amsterdam, Amsterdam, The Netherlands; Translational Medicine, Bristol Myers Squibb, Lawrenceville, NJ, United States; Department of Gastroenterology and Hepatology, Amsterdam UMC, Location University of Amsterdam, Amsterdam, The Netherlands; Tytgat Institute for Liver and Intestinal Research, Amsterdam UMC, Location University of Amsterdam, Amsterdam, The Netherlands; Amsterdam Gastroenterology Endocrinology Metabolism (AGEM), Amsterdam, The Netherlands; Department of Gastroenterology and Hepatology, Amsterdam UMC, Location University of Amsterdam, Amsterdam, The Netherlands; Tytgat Institute for Liver and Intestinal Research, Amsterdam UMC, Location University of Amsterdam, Amsterdam, The Netherlands; Amsterdam Gastroenterology Endocrinology Metabolism (AGEM), Amsterdam, The Netherlands; Department of Gastroenterology and Hepatology, Amsterdam UMC, Location University of Amsterdam, Amsterdam, The Netherlands; Department of ImmunePathophysiology and Surgery, University of Bonn, Bonn, Germany

**Keywords:** ulcerative colitis, B cell pathology, B cell maturation and expansion, mice colitis transfer model, single-cell RNA-sequencing

## Abstract

**Background:**

Ulcerative colitis (UC) is a chronic inflammatory bowel disease characterized by relapsing and remitting mucosal inflammation of the colon. While active UC mucosa is characterized by dysregulated B cell responses and increased B cell and IgG plasma cell populations, targeting CD20-expressing B cells in UC has proven ineffective.

**Methods:**

We conducted an exploratory single-cell transcriptomic analysis of colonic biopsies obtained from UC patients with (*n* = 5) or without (*n* = 5) active inflammation, and non-UC controls (*n* = 4). To explore whether B cells contribute to colitis severity, we transferred various ratios of spleen-derived naive B cells with CD45RB^high^ T cells into severe combined immune deficient mice to induce colitis.

**Results:**

Our analysis identified a distinct subset of naive (*MS4A1*^+^*CD27*^-^*IGHD^+^TCL1A^+^*) B cells that are significantly enriched and present a more matured phenotype in inflamed compared to non-inflamed biopsies from UC patients. Cell-cell communication analysis indicated that naive B cells interacted predominantly with CD4^+^ T cell subsets. In the mice transfer colitis model, co-transfer of naive B cells at a ratio of 1-2 T and B cells, respectively, showed an increased maturation and activity, which led to exacerbation of colitis as measured by weight loss, increased colon density, and histological inflammation.

**Conclusion:**

Our findings suggest that naive B cells expand in actively inflamed mucosa from UC patients and play a pathogenic role in experimental colitis.

## Graphical abstract

**Graphical Abstract:**
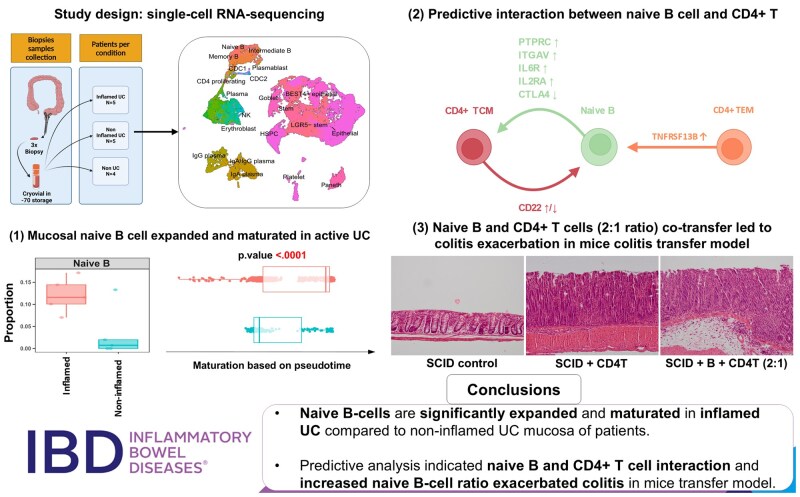

Key Messages **What is already known?**

Depletion of CD20-expressing B cells using rituximab, an anti-CD20, failed to induce significant remission in patients with active moderate-to-severe ulcerative colitis and one case led to severe adverse event.

**What is new here?**

In human colitis mucosa, we identified a *MS4A1^+^ CD27^-^* expanded subset of naive B cells with a more mature phenotype in inflamed mucosa. Naive B cells exacerbated colitis severity when co-transferred with CD45RB^high^ T cells in a murine colitis model.

**How can this study help patient care?**

Selective targeting of pathogenic maturing naive B cells may offer an effective therapeutic strategy for ulcerative colitis, potentially mitigating adverse effects associated with CD20 based pan-B cell depletion approaches.

## Introduction

Ulcerative colitis (UC) is characterized by inflammation of the colonic mucosa, extending from the rectum to the proximal colon.^1^^,^^2^ B cells are key players of the adaptive immune system, which are associated with mucosal inflammation in UC. Within the intestinal mucosa, activated B cells contribute to gut homeostasis by secreting immunoglobulin (Ig) A and IgM antibodies, which are critical in protecting against infections by maintaining the intestinal barrier integrity while simultaneously establishing healthy microbiota.^3-5^ Additionally, anti-inflammatory cytokines are secreted by a regulatory B cell subset and loss of regulatory B cells exacerbate disease activity in mouse models.^6^^,^^7^ Previous studies have shown that B cells accumulate in the inflamed intestinal tissue of IBD patients.^8-10^ The exact role of B cells in inflammatory UC lesions is complex and remains not fully understood. Through single-cell transcriptomic analyses, it was reported that UC patients presented a decreased ratio of IgA/IgG plasma B cells compared to healthy individuals.^10^ Furthermore, it was demonstrated that mucosal IgG and FcR binding elicits a pathogenic Th17 response in UC.^11^ Notably, plasma B cell driven changes were reflected in circulation as evidenced by the expansion of gut-homing β7 plasmablasts, which correlated with UC activity and predicted disease complications.^12^ Furthermore, B cell expansion was found to impair mucosal healing, particularly in damaged areas, and is associated with colitis severity in mouse models.^13^ Notably, Frede et al. (2022)^13^ reported that activated B cells hindered stromal-epithelial crosstalk, and depletion of B cell expansion during mucosal healing accelerates this process. However, depleting B cells with rituximab, a CD20 inhibitor, failed to elicit a significant induction of remission in active moderate-severe UC patients.^14^ This highlights that the role of B cells in UC pathogenesis require further investigation. To this end, we conducted an exploratory study where we characterized the cellular profile of inflamed and non-inflamed colon biopsies through single-cell RNA-sequencing (scRNA-seq). We identified B cells as being the most prominent cell type associated with tissue inflammation state, and found B cells causal in eliciting pathogenic outcomes for colitis through co-transfer of B cells with CD45RB^high^ T cell induced colitis.

## Materials and Methods

### Patient Recruitment and Sampling

Mucosal biopsy samples were obtained from patients during routine ileocolonoscopy at the Department of Gastroenterology and Hepatology of the Amsterdam UMC between December 2020 and April 2021. Patients were aged ≥16 years with an established diagnosis of UC determined through endoscopy and histopathology, where disease activity was scored by a trained gastroenterologist using the Mayo Endoscopic Score (MES) and the Ulcerative Colitis Endoscopic Index of Severity (UCEIS). In total, we acquired samples from 10 UC patients: 5 inflamed UC (MES ≥ 1), 5 non-inflamed UC (MES = 0). Exclusion criteria were ongoing malignancy, a history of colonic dysplasia, or colonic surgery. Control samples were obtained from resection specimens acquired from 4 patients with no established diagnosis of UC (CD, suspicion of rectum carcinoma, trans-anal total mesorectal excision or hemicolectomy), which were obtained from the biobank of the Amsterdam UMC. Detailed patient information is provided in Table S1. Three mucosal colonic biopsies were collected per patient from lesions (inflamed), resolved lesions (non-inflamed), and healthy tissue area (non-UC) and promptly slow-frozen to minus 80 °C in a solution of 10% dimethylsulfoxide (DMSO, Sigma-Aldrich, D8418) and 90% fetal bovine serum (FBS, Serana, S-FBS-SA-015).

This study was conducted with approval by the Medical Ethics Committee Amsterdam UMC, University of Amsterdam (#NL75341.018.20) and in accordance with the principles of the Declaration of Helsinki (version 2013), the Medical Research Involving Human Subjects Act (WMO), International Conference on Harmonization Good Clinical Practice, current European guidelines, the biobank committee of the Amsterdam UMC (178 #A201470) regulations and acts. All participating patients provided written informed consent before study participation.

### Single-Cell RNA-Sequencing Preparation

The biopsy samples were prepared in accordance with previously established protocols.^15^ Samples were thawed in a 37 °C water bath, rinsed with complete medium of RPMI 1640 (Gibco) supplemented with 100 U/mL Penicillin-Streptomycin (Gibco), 2 mM L-glutamine (Capricorn Scientific), 25 µg/mL fungizone (Gibco), and 20 mg/mL gentamicin (Lonza), and gently shaken and centrifuged at 500 g for 5 minutes. The biopsies were subsequently transferred to a 6-well plate, containing a 1.5 mL digestion mixture composed of base complete medium, 0.5 mg/mL Collagenase IV (Sigma-Aldrich), 10 mg/mL DNase, and 2% FBS. The biopsies were carefully dissected into smaller pieces using tweezers and a scalpel, where digestion was carried out at 37 °C for 45 minutes at 160 RPM in a dry shaking incubator. The resultant single-cell suspensions were passed through a 100 µm EasyStrainer^tm^ cell strainer (Greiner Bio-one) and washed with MACS buffer whereupon dead cells were removed by the dead cell removal kit (Miltenyi) following the manufacturer’s instructions. The resultant single cell suspensions were subjected to microscopic examination and cell counting using a cell chamber (Bürker, Marienfeld) diluted in trypan blue at a 1:1 ratio. After removing the supernatant, the cells were concentrated to a final concentration of 1000 cells/µL. Cell viability and concentration were determined using a Countess II FL Automated Cell Counter. The prepared samples were subsequently sent to the Amsterdam UMC Core Facility Genomics for the 10× Genomics Single Cell protocol, utilizing the 10× Genomics Chemistry v3 kit, where after paired ended sequencing was carried out on a NovaSeq6000.

### Single-Cell RNA-Sequencing Data Analysis

Raw reads were aligned to GRCh38 using Cellranger (v7.0.0) (10× Genomics) generating unique molecular identifiers (UMIs) were obtained. Samples were imported separately, processed, and analyzed in the R programming environment (v4.2.1) using Seurat (v4.3.0).^16^^,^^17^ UMI counts were normalized by SCTransform (v) using default parameters. Cells were initially divided into live singlets and debris or dead cells based on the latter presenting a low number of unique genes and a high percentage of mitochondrial reads. Immune cells were annotated through multimodal reference-mapping against a peripheral blood mononuclear cell (PBMC) cellular indexing of transcriptomes and epitopes sequencing (CITE-seq) dataset.^17^^,^^18^ A secondary manual curation was subsequently implemented to annotate the non-immune cell types. The live cells were subsequently characterized as epithelial cells (*EPCAM*^+^*ANPEP*^+^*VIL1*^+^) and the immune (*PTPRC*^+^) cells. The epithelial cells were subdivided into *BEST4*^+^ epithelial cells (*OTOP2^+^BEST4^+^*), goblet cells (*MUC1^+^MUC2^+^*), stem cells (*KCNQ1^+^KCNE3^+^OLFM4^+^LGR5^+^*), *LGR5^-^* stem cells (*KCNQ1^+^KCNE3^+^OLFM4^+^*), and Paneth cells (*CDH1*^+^  *SOX9^+^LYZ^+^*).^10^^,^^19^^,^^20^ The immune cells were initially annotated against a multimodal reference PBMC CITE-seq experiment,^17^^,^^18^ where after manual curation confirmed their identity as T cells (*CD3D^+^*, *CD2*^+^), subdivided into CD4^+^ T cells, *CD8*^+^ T cells, and CD4^-^*CD8*^-^ T cells. CD4^+^ T cells further differentiated into multiple subsets, including CD4^+^ naive T cells (*TCF7*, *CD4*, *CCR7*, *IL7R*, *LEF1*, *MAL,* and *PIK3IP1*), CD4^+^ proliferating T cells (*MKI67*, *TOP2A*, *PCLAF*, *CENPF*, *TYMS*, *NUSAP1*, *ASPM*, *PTTG1*, *TPX2,* and *RRM2*), CD4^+^ central memory T cells (TCM; *IL7R*, *CD4*, *LTB*, *TRAC*, *AQP3*, *LDHB,* and *MAL*), CD4^+^ effector memory T cells (TEM) (*IL7R*, *CCL5*, *GZMK*, *IL32*, *GZMA*, *KLRB1*, *TRAC*, *LTB,* and *AQP3*), CD4^+^ regulatory T cells (*RTKN2*, *FOXP3*, *CD4*, *IL2RA*, *TIGIT*, *CTLA4,* and *IKZF2*). For *CD8*^+^ T cells, we could identify *CD8*^+^ naive T cells (*CD8B*, *CCR7*, *RGS10*, *LINC02446*, *LEF1*, *CD8A,* and *OXNAD1*), *CD8*^+^ proliferating T cells (*MKI67*, *CD8B*, *TYMS*, *TRAC*, *PCLAF*, *CD3D*, *CLSPN*, *CD3G*, *TK1,* and *RRM2*), *CD8*^+^ central memory T cells (*CD8B*, *ANXA1*, *CD8A*, *LINC02446*, *YBX3*, *IL7R*, *TRAC*, *NELL2,* and *LDHB*) and *CD8*^+^ effector memory T cells (*CCL5*, *GZMH*, *CD8A*, *TRAC*, *GZMK*, *CST7*, *CD8B,* and *TRGC2*). *CD4*^-^*CD8*^-^ T cells encompassed double negative T cells (DNT; *PTPN3*, *MIR4422HG*, *CAV1*, *GZMA*, *MYB*, *FXYD2*, *GZMK,* and *AC004585*.1), γδ T cells (GDT; *TRDC*, *TRGC1*, *TRGC2*, *KLRC1*, *NKG7*, *TRDV2*, *KLRD1,* and *KLRG1*) and mucosal associated invariant T cells (MAIT; *KLRB1*, *NKG7*, *GZMK*, *IL7R*, *SLC4A10*, *GZMA*, *CXCR6,* and *NCR3*).

Among B cells, we distinguished 3 subgroups with additional highlights of (non-) canonical marker(s) including multimodal reference-mapping^18^: naive B cells (*IGHD*, *TCL1A,* and *CD27*^-^), intermediate B cells (*TNFRSF13B*, *LINC01857,* and *CD27*^+/-^) and memory B cells (*TEX9*, *LINC01781,* and *CD27*^+^) (Figure S3 and Table S2). Among plasma B cells, we could divide into 4 subgroups that are plasmablasts (*TYMS*, *SHCBP1*, *TK1*, *KNL1*, *ASPM*, *TPX2*, *RRM2,* and *BIRC5*), plasma cells (*MZB1*, *JCHAIN*, *DERL3*, *CD27*, *ITM2C*, *TNFRSF17*, *TXNDC5*, *POU2AF1,* and *CD79A*), immunoglobulin G plasma cells (IgG plasma; *MZB1*, *JCHAIN*, *DERL3*, *CD27*, *ITM2C*, *TNFRSF17*, *TXNDC5*, *POU2AF1*, *CD79A*, *IGHG1*, *IGHG2*, *IGHG3,* and *IGHG4*), immunoglobulin A plasma cells (IgA plasma; *MZB1*, *JCHAIN*, *DERL3*, *CD27*, *ITM2C*, *TNFRSF17*, *TXNDC5*, *POU2AF1*, *CD79A*, *IGHA1,* and *IGHA2*) and multiplet IgA/IgG plasma cells (*MZB1*, *JCHAIN*, *DERL3*, *CD27*, *ITM2C*, *TNFRSF17*, *TXNDC5*, *POU2AF1*, *CD79A*, *IGHA1*, *IGHA2*, *IGHG1*, *IGHG2*, *IGHG3*, *IGHG4*).

Among other lymphoid cells, we identified Natural killer cells (NK; GNLY, *TYROBP*, *NKG7*, *FCER1G*, *GZMB*, *TRDC*, *PRF1*, *FGFBP2*, *SPON2,* and *KLRF1*), NK proliferating cells (*MKI67*, *KLRF1*, *TYMS*, *TRDC*, *TOP2A*, *FCER1G*, *PCLAF*, *CD247*, *CLSPN,* and *ASPM*) and innate lymphoid cell (ILC; *KIT*, *TRDC*, *KLRB1*, *TNFRSF18*, *TNFRSF4,* and *IL1R1*).

Dendritic cells were also identified, consisting of conventional dendritic cell 1 (CDC1; *NDRG2*, *CLEC9A*, *DNASE1L3*, *C1orf54*, *IDO1*, *CLNK*, *CADM1*, *FLT3*, *ENPP1,* and *XCR1*), conventional dendritic cell 2 (CDC2; *NDRG2*, *FCER1A*, *CLEC10A,* and *CD1C*) and plasmacytoid dendritic cell (*CLEC4C*, *SCAMP5*, *PTCRA*, *SCT*, *SHD*, *LILRA4,* and *LRRC26*).

Monocytes were also identified (*CTSS*, *FCN1*, *NEAT1*, *LYZ*, *PSAP*, *S100A9*, *AIF1*, *MNDA*, *SERPINA1,* and *TYROBP*), which could be subdivided in classical monocyte (*S100A8*, *S100A12*, *VCAN*, *FCN1*, *FPR1,* and *SELL*) and non-classical monocyte (*FCGR3A*, *SELL^-^,* and *S100A12^-^*).

Additionally, various other cell types were identified, which encompassed of hematopoietic stem and progenitor cell (HSPC; *CYTL1, GATA2*, *SMIM24*, *AVP*, *MYB*, and *LAPTM4B*) and platelet (*PPBP*, *GNG11*, and *CAVIN2*).

### Mice and Cell Isolation

Female C.B 17 SCID (C.B 17/lcrHsd-Prkcd<scid>) mice and wild-type BALB/c (BALB/cOlaHsd) mice were ordered from Harlan (Boxmeer, The Netherlands). CD4^+^CD45RB^high^ cells were isolated and sorted using fluorescence-activated cell sorting (FACSorting), following established procedures.^21^ Splenic B cells were isolated through magnetic sorting (negative selection) employing a B cell isolation kit (Miltenyi Biotec, cat#130-090-862). In brief, a single-cell suspension was prepared by passing spleens from wild-type BALB/c mice through a cell strainer. Erythrocytes were removed using erythrocyte lysis buffer, followed by negative depletion of macrophages, T cells, and residual erythrocytes using magnetic beads. Flow cytometry confirmed that resulting cell suspensions were >98% B cell positive, as determined by *CD45R* (1:200, B220-monoclonal antibody-PE, RA3-6B2, eBiosciences). Mesenteric lymph nodes (MLNs) were dissected and crushed through 70 um filters. After 15 minutes, fixation in 1% PFA cells were stained with anti-B220-PE (Immuno source, 12-0452-83), permeabilized with 0,5% Saponine, and stained with anti-IgA-FITC (BD Pharmingen, 559354) and analyzed using a FACS Fortessa (BD) and FlowJo software (Treestar Inc., Ashland, OR).

### Immunohistochemistry

Sections (5 µm) were deparaffinized in xylene and rehydrated. Endogenous peroxidase was blocked using 0.3% H_2_O_2_ in methanol for 30 minutes. For antigen retrieval slides were cooked at 100 °C for 10 minutes in EDTA pH 9.0. After antigen retrieval slides were blocked in phosphate-buffered saline (PBS) with 1% bovine serum albumin (1%BSA/PBS) for 30 minutes at room temperature (RT), followed by incubation overnight at 4 °C with a rabbit-anti-mouse-CD19 (Abcam, ab245235). Antibody binding was visualized using Powervision horseradish-peroxidase-labeled secondary antibodies from Immunologic and diaminobenzidine (Sigma-Aldrich) for substrate development. All sections were counterstained with Mayer’s hematoxylin (Sigma-Aldrich).

### Induction of Colitis and Sample Collection

Colitis was induced by transferring 4.0 × 10^5^ CD4^+^CD45RB^high^ cells to SCID mice via intraperitoneal injection. Cells were either transferred without B cells or supplemented with the following number of B cells: 2.0 × 10^5^ (2 T to 1B cell ratio), 4.0 × 10^5^ (1 T to 1B cell ratio), or 8.0 × 10^5^ (1 T to 2B cell ratio). Mice were sacrificed via CO_2_ asphyxiation and blood samples were collected by cardiac puncture. The large intestine was excised, longitudinally opened and cleaned of feces. Disease activity was assessed using the disease activity index (DAI), which considers stool consistency, the presence of blood and tissue edema, by blinded evaluation. The length of the large intestine, measured in a relaxed position without stretching, was used to calculate colon density (mg/cm). The intestine was thoroughly washed with PBS and longitudinally divided into 2 sections. One section was processed as a “swiss roll,” routinely embedded in paraffin, and 5 µm slides were prepared and stained with hematoxylin and eosin (H&E) using standard protocols. The mouse colitis histology index (MCHI), scored from 0 to 22, was determined by a blinded observer, as previously described.^21^ The other section was frozen at −80 °C, and homogenate samples were later prepared using a Precellys tissue homogenizer with cell lysis buffer and protease inhibitors. After centrifugation, the supernatant was collected and protein concentrations were determined using a BCA kit (ThermoFisher Scientific, Waltham, United States). Ig levels were determined in serum and colon homogenates by IgG, IgA and IgM Ready Set-Go ELISAs (eBiosciences, cat nrs: 88-50450-77, 88-50400-77, 88-50400-77, and 88-50470-77), according to manufacturer’s instructions.

### Quantification and Statistical Test Analyses

To discern differences in the cell types of interest, immune cells were filtered into subsets from the SeuratObject for subsequent differential abundance analysis. This analysis was performed using the propeller package, leveraging biological replication to detect statistically significant differences in cell type proportions among the selected groups.^22^ The Slingshot package was utilized for inferring the developmental trajectory and subsequent statistical analysis of maturation status of a cell, facilitating the lineage and pseudotime inference.^23^ In short, slingshot reconstructs lineages by inferring a global lineage structure and then calculating the progression or trajectory of each cell along each lineage which is termed the pseudotime. Importantly, the pseudotime does not represent real time but rather a heuristic inferred from the relative progression or maturation of cells. By using slingshot, we can order cells from early to late stages providing insight into differentiating cells in the absence of actual time-course experiments. Following pseudotime quantification, T-test/Mann-Whitney U test was conducted to assess statistical differences between the inflamed and non-inflamed group in R.^16^ Furthermore, differential expression of naive B cells was determined by the package DESeq2, which provides methods to test differential expression by the use of negative distributions on the normalized raw gene expression count.^24^ Ambient RNA contamination was addressed using the EmptyDrops method.^25^ Genes flagged as potentially contaminated by this approach were excluded from subsequent differential expression analyses to minimize false positives due to background RNA. Subsequently, cell–cell communication analysis was performed using the MultiNicheNet package on the immune population with naive B cells as main interest, which is a novel framework that analyze multi-sample multi-condition single-cell transcriptomics data.^26^ This tool identifies differentially expressed active ligand-receptor pairs between the groups of interest and predicts the interaction of these selected pairs based on the expression of the associated downstream genes. We followed the suggested vignette (MultiNicheNet—comprehensive tutorial—Condition A vs Condition B vs Condition C) of MultiNicheNet as suggested in their GitHub. For the mice experiments, outputs of colon density, MCHI, DAI and immunoglobulin concentrations were tested for normality and lognormality tests with subsequent Mann-Whitney or unpaired T-tests combined with Bonferroni correction by GraphPad Prism version 9.5.1 for Windows, GraphPad Software, Boston, MA, United States, www.graphpad.com.

### Public Data

Single-cell RNA-sequencing datasets published by Martin et al., Smilie et al., Uzzan et al. and Kong et al. were acquired from the Gene Expression Omnibus.^10^^,^^12^^,^^27^^,^^28^ Raw reads were aligned to GRCh38 using Cellranger (v7.0.0) (10× Genomics) generating UMIs were obtained. Samples were imported separately, processed, and analyzed in the R programming environment (v4.2.1) using Seurat (v4.3.0).^16^^,^^17^ UMI counts were normalized using SCTransform (v) using default parameters. Cell subset annotations were manually curated and matched to our own dataset for comparability purposes as described above. The same analysis was performed using the propeller package to detect statistically significant differences in cell type proportions among the selected groups, differential expression analysis using the DESeq2 package and pseudotime analysis using the Slingshot package.^22-24^

## Results

### B Cells Are Enriched in Inflamed UC Mucosa

**Figure 1.**
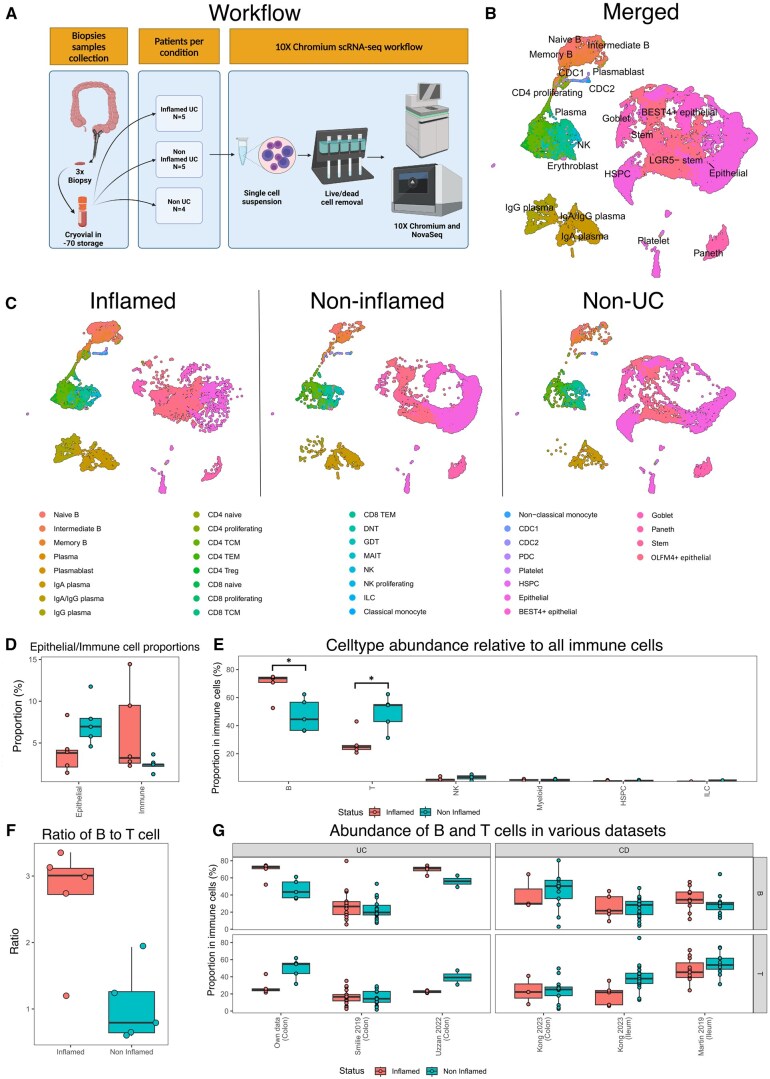
Higher abundance B cells in UC-derived tissues when comparing inflamed with non-inflamed tissues. (A) Single-cell RNA-sequencing (scRNA-seq) processing workflow of biopsies obtained from inflamed (*n* = 5), non-inflamed (*n* = 5) and non-UC (*n* = 4) patients. Created in BioRender. Fung, I. (2025) https://BioRender.com/l4mvyx2. Uniform manifold approximation and projection (UMAP) of all measured cells (B) annotated to their respective cell type and (C) split by inflammatory group. (D) Boxplots of the proportion immune and epithelial cells relative to all live singlets cells show that inflamed biopsies present a larger immune cell population compared to non-inflamed biopsies. (E) Boxplots of the proportion immune cells show that inflamed biopsies exhibit a significantly higher proportion of B cells and a lower proportion T cells compared to non-inflamed biopsies. (F) Boxplots of the B to T cell ratio show a higher ratio of B to T cell ratio in inflamed biopsies compared to non-inflamed biopsies. (G) Boxplots of the B and T cell abundance show a higher proportion of B cells and a lower proportion T cells relative to all immune cells in Smilie et al. (UC) and Uzzan et al. (UC), Martin et al. (CD), and Kong et al. (CD) compared to non-inflamed biopsies in UC, but not in CD.

Colon biopsies of unpaired samples were collected from inflamed tissue (*n* = 5) and non-inflamed tissue (*n* = 5) from UC patients. An additional set of colon biopsies was obtained from non-UC patients (*n* = 4) as control (Table 1 and Table S1). Colon biopsies were enzymatically digested, yielding single cell RNA-sequencing data of 44.792 cells after quality control that were included in subsequent analyses (Figure 1A). As a first step, we identified *EPCAM*^+^ epithelial cells and *PTPRC*^+^ immune cells (Figure S1A). Epithelial cells were further annotated into *BEST4^+^* epithelial, *MUC1^+^* and *MUC2^+^* goblet, *LYZ^+^* Paneth, and *LGR5^+^* stem cells. Immune cells were further categorized into *MS4A1^+^* (CD20) or *JCHAIN*^+^ B cells, *CD3D^+^* T cells, *NKG7*^+^ NK cells, and various myeloid lineages such as *S100A8^+^* classical monocytes and *FLT3^+^* dendritic cells (Table S2 and Figure 1B). All the main lineages were found in both UC and non-UC samples, with no significant differences in abundance in immune subsets between non-inflamed compared to non-UC (Figure 1C and Figure S1B,S1C). Focusing on UC patients, we found a larger immune cell fraction and a diminished epithelial fraction when comparing biopsies from inflamed regions with biopsies from non-inflamed regions, although not significant (*P* = .082) (Figure 1C,1D), reflecting an numerical increase of immune cell infiltration in line with the inflamed status of the tissue. Relative to all immune cells, the most differentially abundant cell types were *MS4A1^+^* B (*P* = .047) and *CD3D^+^* T cells (*P* = .047), which were enriched in inflamed and non-inflamed tissue, respectively (Figure 1E). B cells appear to be more enriched than T cells in inflamed tissues with a B/T cell ratio of ∼3-1 compared to the ∼1-1 ratio observed in non-inflamed tissues, which could be validated in the dataset of Uzzan et al. (2022),^12^ although this could not be validated in the dataset of Smillie et al. (2019)^10^ (Figure 1F and Figure S2A,S2B). Subsequent analysis of previously published datasets showed that the elevated B cell abundance corroborates earlier observations in UC,^10^^,^^12^ while these differences were not present in either colonic^27^ or ileal^27^^,^^28^ biopsies from patients with Crohn’s disease (CD) (Figure 1G).

**Table 1.** Patient characteristics of inflamed and non-inflamed UC at the time of sampling included in the scRNA-seq.

Demographic	Inflamed (*n* = 5)	Non-inflamed (*n* = 5)	*P*-value
**Age (years), median [IQR]**	23 [20.5-44.5]	42 [35.5-61]	.047
**Female, *n* (%)**	4 (80)	2 (40)	.221
**Origin, *n* (%)**			1
** - Caucasian**	4 (80)	4 (80)	
** - Surinamese**	1 (20)	1 (20)	
**Clinical**			
**Disease duration (years), median [IQR]**	3 [1.5-17]	14 [10-16.5]	.117
**Disease extent, *n* (%)**			.817
** - Proctitis**	0 (0)	1 (20)	
** - Left-sided**	3 (60)	1 (20)	
** - Pancolitis**	2 (40)	3 (60)	
**≥2 Previous failed classes of advanced therapies, *n* (%)**	2 (40)	2 (40)	1
**Full MAYO score, median [IQR]**	8 [6-9]	1 [0-3]	.008
**Endoscopic MAYO score**			.008
** - 0-1**	0 (0)	5 (100)	
** - 2**	5 (100)	0 (0)	
**UCEIS, median [IQR]**	6 [4.5-6.5]	3 [3-4.5]	.049
**Fecal calprotectin (µg/g), median [IQR]**	1290 [124-2554]	82 [10-575]	.047
**Smoking status, *n* (%)**			.221
** - No**	4 (80)	2 (40)	
** - Former**	1 (20)	3 (60)	

Overview of demographic and clinical aspects of the included UC patients. IQR, interquartile range.

The red colored values are values below p = 0.05, meaning signifcant.

### Naive B Cells Are Enriched and Express a More Matured Phenotype in Inflamed UC Mucosa

**Figure 2.**
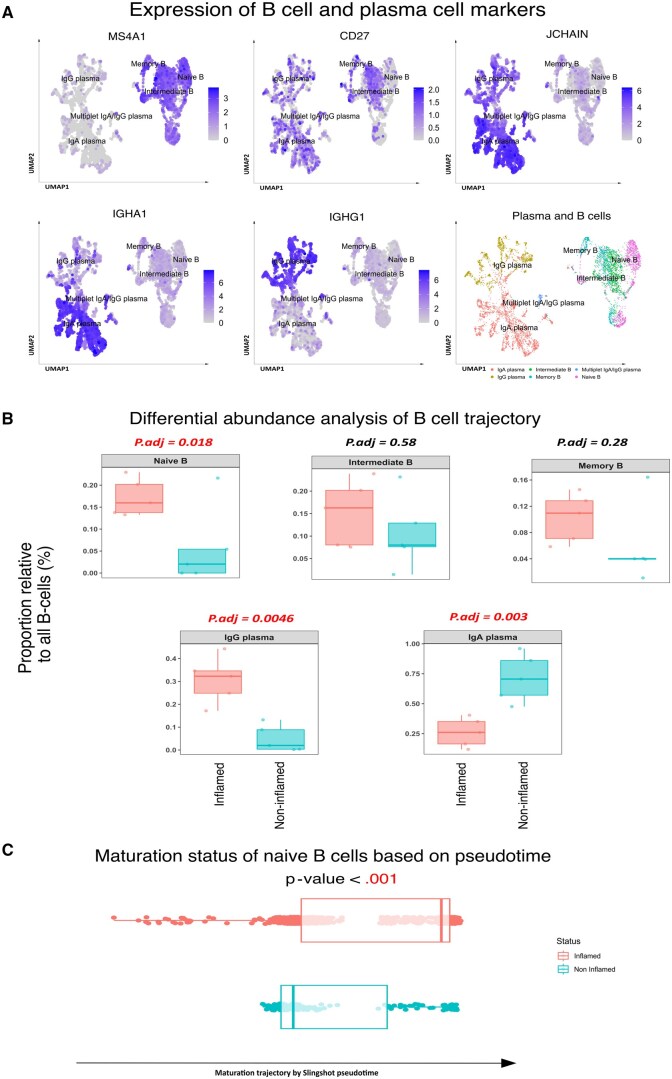
Enriched naive B cells exhibit higher maturation in inflamed UC. (A) UMAPs of B cell and plasma markers delineating the B cell trajectory. *MS4A1* (*CD20*) are broadly expressed by B cells, while *CD27* expression is absent in naive B cells and gradually increases from intermediate B cell to memory B cell. *JCHAIN* is mainly expressed in the plasma, which can be further divided into IgA (*IGHA1*) and IgG (*IGHG1*) plasma cells. B and plasma cell annotations are shown in the UMAP overview. (B) Differential abundance analysis reveals a significantly higher abundance of naive B cells and IgG plasma cells in inflamed and IgA plasma cells in non-inflamed UC B cell trajectory. (C) Naive B cells appear to be more mature based on the pseudotime analysis in inflamed UC compared to non-inflamed UC (*P* < .001).

Focusing on the B cell lineage, B cells and plasma cells were found to cluster separately and could be distinguished based on their mutually exclusive expression of *MS4A1* and *JCHAIN* on B and plasma cells, respectively. B cells could be further divided into naive (*CD27*^-^  *IGHD^+^TCL1A^+^*), intermediate (*CD27*^+^  *LINC01857*^+^*TNFRSF13B^+^*), and memory (*CD27*^++^  *TEX9^+^LINC01781^+^*) B cells, as defined using the PBMC reference dataset,^18^ whereas plasma cells could be separated into IgA- and IgG-expressing plasma cells based on their expression of *IGHA1* or *IGHG1* (Figure 2A and Figure S3). Comparing B cell and plasma subsets between inflamed and non-inflamed UC tissues, relative to the entire B cell lineage, indicated a significantly higher abundance of naive B cells (*P* = .018) and IgG plasma cells (*P* = 4.6 E−03) in the inflamed regions, while IgA plasma cells (*P* = 3 E−03) show a significantly higher abundance in non-inflamed regions (Figure 2B). The significant higher abundance of naive B cells could be confirmed in the dataset of Uzzan et al. (2022)^12^ (*P* = .002), however, this was not the case in the dataset of Smillie et al. (2019)^10^ (*P* = .91) (Figure S2C and S2D). When interrogating the abundance of naive B cells, we note no significant differences in differential abundance when comparing non-inflamed UC and non-UC (Figure S4A). Subsequently, through trajectory analyses, naive B cells in inflamed UC tissues presented with a more mature phenotype compared to those in non-inflamed tissues of UC patients based on pseudotime^23^ (*P* < 1.0 E−03, Figure 2C). We could validate significant a more mature phenotype of naive B cells in Smillie’s datasets,^10^ while it is not significant, showing the similar trend in Uzzan’s dataset^12^ (*P* < .001 in Smillie’s dataset, *P* = .14 in Uzzan’s dataset, Figure S2E,S2F). Notably, non-inflamed tissue B cells presented a more mature phenotype compared to non-UC group, despite being less mature compared to inflamed tissue of UC patients (Figure S4B).

### Naive B Cells Predominantly Interact with CD4+ T Cells as Stimulatory Signaler and Receiver

**Figure 3.**
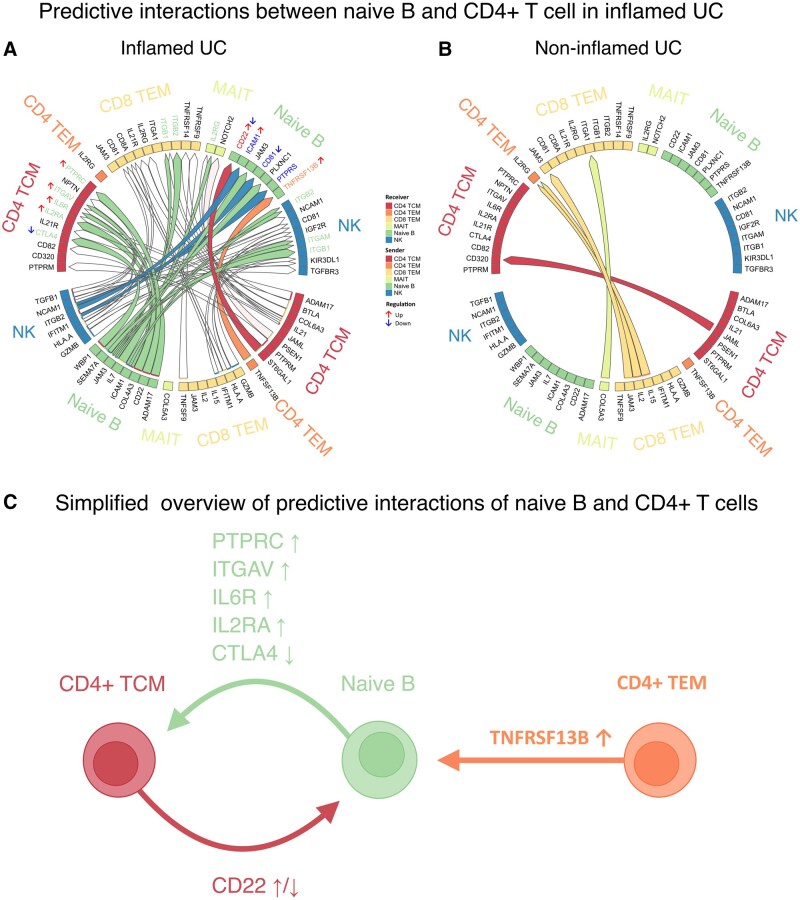
Predictive interactions predominantly between naive B cells and CD4^+^ T cells. (A,B) Circle plots denoting receptor-ligand pairings with a higher aggregated prioritization score (APS) in UC biopsies based on MultiNicheNet analysis. Connections between receptors and ligands are grouped and colored for the cell-type expressing the ligand. APS is based on differential expression of receptor, ligand and downstream signaling genes. Additionally, higher score is assigned to interactions specific for experimental groups, in this case inflamed and non-inflamed tissues. Plots are split for interactions found significantly more in inflamed (A) or non-inflamed (B) biopsies when compared to one another. Ligand-receptor pairings with the 50 highest APS are shown. Of the pairings with the highest score, ligand-receptor pairs were predominantly identified in inflamed UC, where naive B cells primarily acted as interacting senders with CD4+ central memory T cells (TCM) and as receivers with CD4^+^ T cell subsets and NK cells. No significant interactions involving naive B cells as senders or receivers were identified in non-inflamed UC. Red arrows next to gene names indicate stimulatory signals, while blue arrows denote regulatory or inhibitory signals. For readability arrows denoting ligand-receptor pairings not involved with naive B cells were not filled in. (C) Simplified overview of interactions between naive B cell and CD4^+^ T cell subsets. *PTPRC* (CD45), *ITGAV*, *IL6R*, *IL2RA,* and *CTLA4* of CD4^+^ TCM (receiver) were predicted to be activated by naive B cells (sender). *TNFRSF13B* and *CD22* in naive B cells (receiver) were predicted to be activated by CD4^+^ TEM and TCM (senders), respectively.

To understand the potential role of the naive B cells in inflamed UC, we conducted a differential ligand-receptor analysis using MultiNicheNet to identify which immune cells were tentatively communicating with the naive B cells.^26^ To identify the most present ligand-receptor pairs, the top 50 most differentially expressed ligand-receptor pairs were selected. We identified significant interactions between naive B cells and CD4^*+*^ central memory T cell (TCM), CD4^*+*^ effector memory T cells (TEM), CD8*^+^* TEM, MAIT, and natural killer (NK) cells in inflamed UC compared to non-inflamed UC (Figure 3A). Notably, interactions between naive B cells and other immune cells were not increased in the top 50 ligand receptor pairs in non-inflamed tissues compared to inflamed tissues of UC patients (Figure 3B). Naive B cells were found to interact with CD4^+^ TCM cells, indicating processes such as activation, adhesion, proliferation, and differentiation towards T helper (Th) 2 or Th17 phenotypes through the receptor genes *PTPRC*, *ITGAV*, *IL6R*, *IL2RA*, and *CTLA4*.^29-33^ Furthermore, naive B cells indicated to interact with CD8^+^ TEM cells and NK cells via integrin β2 (*ITGB2* and *ITGAM*) and *ITGB1*, which could facilitate adhesion and cell surface signaling.^34^^,^^35^ Additionally, interactions involving *IL2RG* in CD8^+^ TEM cells were observed, indicating potential involvement in interleukin signaling pathways. Conversely, as receivers, naive B cells were indicated to be stimulated by CD4^+^  *TCM* and *TEM* cells through interactions with *CD22* and *TNFRSF13B*. Notably, *CD22* is implicated in B cell modulation, homeostasis and inhibition of B cell receptor (BCR) signaling, while *TNFRSF13B* influences B cell survival and development (Figure 3C).^36^^,^^37^ In summary, our findings suggest extensive communication between CD4^+^ T cells and naive B cells in inflamed UC, driving processes of differentiation, maturation, activation, and other immune responses, while interactions with other cell types are less present.

### Expansion and Maturation of Naive B Cells Contributes to Colitis Severity

**Figure 4.**
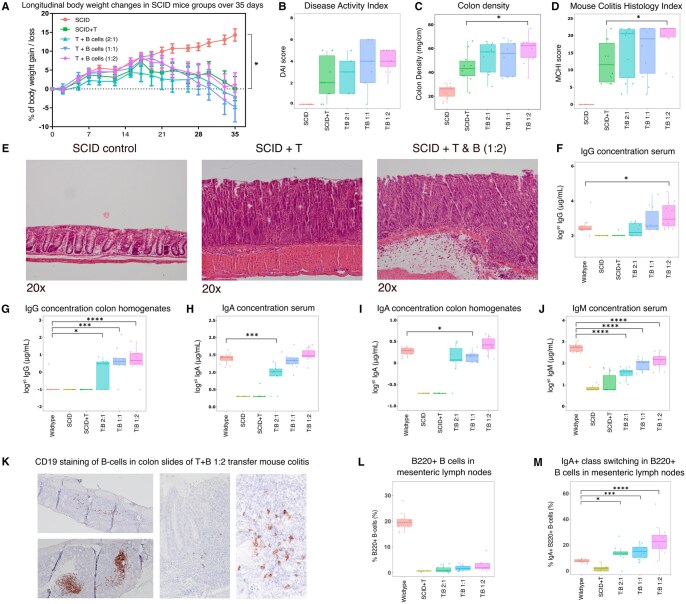
Naive B cell expansion and maturation exacerbates colitis severity in SCID mice. (A) Body weight changes were observed for 35 days of SCID mice. SCID control showed a body weight gain, while SCID+T cell control and T: B ratios resulted in significant body weight loss. (B) A trend of increased DAI scores was observed in all T: B ratios, although not statistically significant. (C,D) Ratios of 2T to 1B cells and 1T to 1B cell presented trends of increased colon density and MCHI, with a significant increase at a ratio of 1T to 2B cells. (E) H&E staining of SCID, SCID+T cell control and SCID+T : B (1T to 2B cells). SCID presented a normal mucosa of the colon, which was disrupted in the SCID+T cell control. SCID+T : B (1T to 2B cells) exhibited further aggravation of mucosal disruption compared to SCID+T cell control. (F-J) Immunoglobulin levels (IgG, IgA and IgM) were measured in serum and colon homogenates. Significant increases in IgG were observed in the serum of the ratio 1T to 2B cells and in colon homogenates across all T : B ratios. No increase was observed for IgA levels, while decrease of IgM levels was observed. (K) CD19 immunohistochemistry staining of 1T to 2B transfer colitis mice colon slide. (L) B220+ B cells population were measured in MLNs. B220+ B cells were found to be more abundant in wildtype compared to the T : B groups. (M) IgA activity was measured of the B220+ B cells. IgA activity was significantly higher in all T : B ratios, specifically in a 1T to 2B ratio.

Having established a theoretical framework through which T and B cells communicated under inflammatory conditions in UC, we next investigated whether their functional properties contributed to colitis in a mouse model. Resting (naive) B cells and CD45RB^high^ T cells were added in 3 different ratios (1T-1B, 2T-1B, and 1T-2B) and injected intraperitoneally into C.B 17 SCID host mice to induce colitis. Over the course of 5 weeks, SCID control mice exhibited consistent weight gain, whereas all T cell-inoculated mice experienced significant weight loss between days 17 and 35, indicative of colitis development. The addition of B cells to T cell-inoculated groups did not significantly alter the weight loss trajectory (Figure 4A). Notably, a trend was observed towards an increased disease activity index (DAI) with co-transfer of more B cells, albeit statistically non-significant. (Figure 4B). In contrast, SCID mice receiving 1T cells and 2B cells ratio, similar to the ratio observed in the patient-derived inflamed UC biopsies (1T : 2-3B cells ratio, Table S3), exhibited significantly exacerbated colitis compared to those receiving T cells alone as evidenced by increased colon density and a higher MCHI.^21^ While other groups with added B cells indicated a similar trend, the differences were not statistically significant compared to the T cell-only group. (Figure 4C,D). Histological examination substantiated the heightened disease activity in the group with 1T-2B ratio, evident through disruption of the colonic mucosa compared to both the SCID control and T cell-only group (Figure 4E). Determination of the Ig levels in serum and colon homogenates indicated significantly elevated IgG levels in circulation along with the intestine of mice that received 1T-2B cells when compared to non-diseased wild type BALBc mice (donor mice of the transferred T and B cells) (Figure 4F,G). In contrast, the colonic and serum IgA levels were not elevated, while circulating IgM levels were significantly decreased. (Figure 4H,J). To localize the B cells post-transfer, we observed clear infiltration of CD19^+^ B cells within inflamed colon tissue through immunohistochemistry, indicating that these cells localize to the colon in the 1T-2B cells group (Figure 4K). Thereafter, FACS analysis of mesenteric lymph nodes (MLNs) identified that the B cells were found systemically post-transfer. While the total number of B220^+^ B cells was lower in T : B cell groups compared to wildtype controls (Figure 4L), IgA^+^ class-switching was most notable in mice inoculated with T : B cells (Figure 4M). Notably, IgA concentrations in serum were similar between wildtype and T + B 1:2 ratio (Figure 4H), despite the lower total B220^+^ B cell count in the latter, suggesting that the IgA^+^ class-switched B cells in the 1T-2B cells group are functionally more active by compensating IgA concentrations. Collectively, these results indicate that CD19^+^ B cells not only infiltrate the colon but also localize systemically and undergo maturation via class-switching during colitis progression, and that this process is closely linked to disease severity in vivo. In summary, our findings highlight the potential of naive B cells, especially when present in a 1T-2B ratio, to exacerbate colitis disease activity in the SCID colitis mouse model.

## Discussion

Through single cell profiling and preclinical models of colitis, we demonstrate that UC-associated colonic inflammation is characterized by an enlarged B cell compartment, primarily representing naive B cells, corroborating earlier observations.^12^ Additionally, these UC-associated naive B cells display a more mature phenotype in inflamed biopsies compared to non-inflamed biopsies evidenced by differentially expressed genes and pseudotime analyses determined in silico. These naive B cells expanding in active UC mucosa are predicted to interact predominantly with CD4^+^ T cells based on receptor-ligand analysis. Furthermore, we observed that *CD40*-*CD40LG* and other T and B cell interactive genes are expressed in both T and B cells, indicating a possible canonical T cell mediated activation of B cell (Figure S5).^38^ Importantly, we demonstrate in a colitis mouse model that excessive naive B cells ultimately lead to increased disease severity.

Interestingly, our co-transfer of B and T cells mouse colitis model resulted in more severe colitis along with elevated IgG levels, similar to what we observed in our active UC patients with significantly increased IgG plasma cells. The elevated IgG levels were associated with increased disease activity, both macro and microscopically. While acknowledging the limited translation of murine models to humans, these findings suggest that plasma B cells may contribute to the pathogenesis of UC, possibly originating from a subset of maturing naive B cells in inflamed tissues. Expansion of naive B cells and IgG plasma cells in UC-derived inflamed tissue was reported to be associated with an increased abundance of short-lived plasma cells.^12^ This B cell dysregulation seems specific for UC rather than IBD as a whole as analysis of public datasets showed that elevated B cell abundance was found in UC, but not in ileal nor colonic biopsies obtained from CD patients.^10^^,^^12^^,^^27^^,^^28^

While previous attempts have been made to target B cells in UC using anti-CD20 monoclonal antibody rituximab, this did not confer any clinical benefits in UC patients and even led to severe adverse event.^14^^,^^39^ We speculate that rituximab may fail to ameliorate symptoms is related to the fact that plasma cells, which do not express CD20, escape depletion and remain active (Figure S6).^40^ Earlier research described how distinct B cell subpopulations exert differential effects on disease pathogenesis in a context-dependent manner. In experimental autoimmune encephalitis, a model for multiple sclerosis, depletion of B cells by rituximab resulted in either exacerbation or improvement of symptoms, depending on whether depletion was performed prior to or after disease onset, demonstrating the duality of B cells.^41^ In collagen induced arthritis, a complete lack of B cells abrogates disease development, but depletion of a specific subset of IL-10 producing B cells results in increased disease severity.^42^ The regulatory role of B cells may be involved in induction, or support of regulatory T cells, as was shown in vitro.^42^ In murine models of intestinal inflammation, a population of CD1d^+^CD5^+^ regulatory B cells has been described as being protective against colitis development through various mechanisms, including the enhancement of regulatory T cell function via cytokines such as IL-10 and TGF-β.^43^ Together, these observations provide context for understanding rituximab’s inefficacy in altering UC disease course and underscore the necessity for a more tailored approach in targeting pathogenic B cells, while preserving regulatory/protective B cells.

It should be noted that our study was cross-sectional in design and we can therefore not provide evidence about the pathogenicity of B cells. Nonetheless, we speculate that naive B cell differentiation is likely involved in pathogenicity of UC, but may not confer pathogenicity in isolation as demonstrated through both in situ and in vivo analyses. Notably, our dataset indicates that naive B cells in UC patients exhibit enhanced maturation and increased abundance. This expansion of maturing and activated B cells correlates with exacerbated colitis severity in vivo, particularly after inoculation at a 1T : 2B cell ratio. These findings align with emerging evidence from systemic lupus erythematosus (SLE) research, where pathogenicity has been linked to maturing naive B cells. Specifically, deep B-cell depletion via CD19 chimeric antigen receptor (CAR) T-cell therapy in SLE results in the re-emergence of naive B cells expressing IgD and IgM, reflecting the selective elimination of mature naive B cells and the persistence of an immature, non–class-switched B-cell compartment.^44^ Interestingly, this further aligns with recent findings by Canales-Herrerias et al. (2024)^45^ that describes the effects of vedolizumab, an anti-α4β7 monoclonal antibody, on both intestinal and peripheral B cell populations in UC patients. In responders, vedolizumab treatment was associated with a significant reduction in naive B cells within gut-associated lymphoid tissue (GALT), alongside decreases in circulating plasmablasts and short-lived plasma B cells. Together, these data underscore the critical role of naive B cell maturation dynamics in pathogenesis and highlight therapeutic potential by targeting pathogenic naive B cells.

Expanding upon this study, future research should focus on the longitudinal monitoring of naive B cells during B and T cell transfer in SCID mice, building upon the findings of this study. Carefully tracking the maturation process of naive B cells over time both in the presence and absence of T cells may provide new insights into how these cells and their interactions with other immune cells contribute to pathogenic mechanisms that exacerbate colitis.

Taken together, our findings reveal the abundance of naive B cells in active human UC and their capacity to cause colitis in murine colitis models. This work underscores the importance of investigating diverse B cell subsets, which likely exert distinct effects in a context-dependent manner. Elucidating the developmental trajectories and functional mechanisms of these B cell populations may pave the way for novel, targeted therapies that mitigate pathology while preserving essential regulatory functions in UC and potentially other (auto)inflammatory diseases.

## Supplementary Material

izaf275_Supplementary_Data

## Data Availability

The data underlying this article are available in Zenodo, at https://doi.org/10.5281/zenodo.14236827.
