# Supplementary figures and images for: Mucosal B Cell Expansion and Maturation Contribute to Colitis Pathogenesis
Source: Inflamm Bowel Dis. 2025 Nov 20;32(2):290–302. doi: 10.1093/ibd/izaf275 (PMC12857420; doi:10.1093/ibd/izaf275)

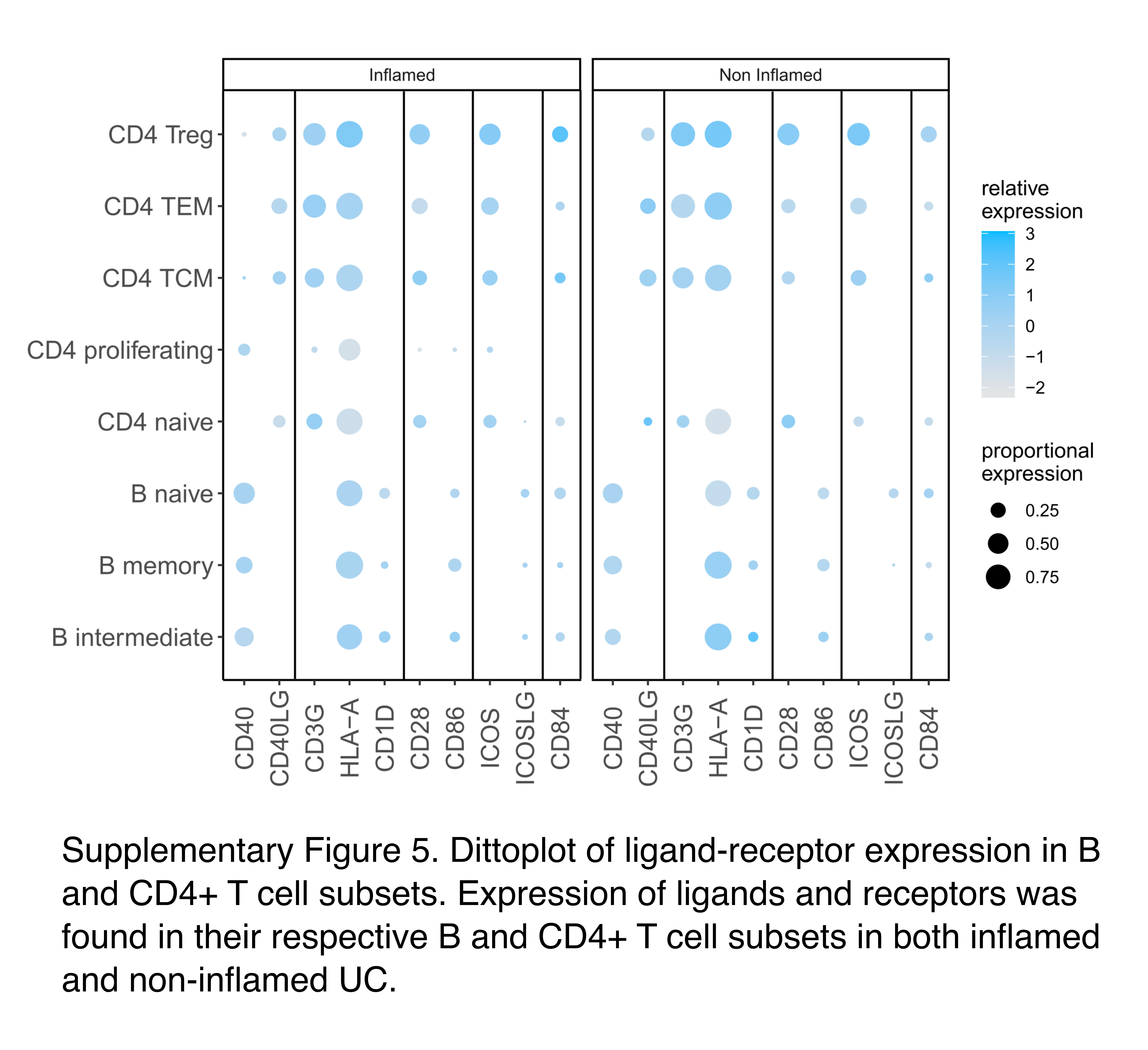

Supplement: izaf275_Supplementary_Data [file izaf275_supplementary_data.zip › Supplementary Figure 5.tiff]

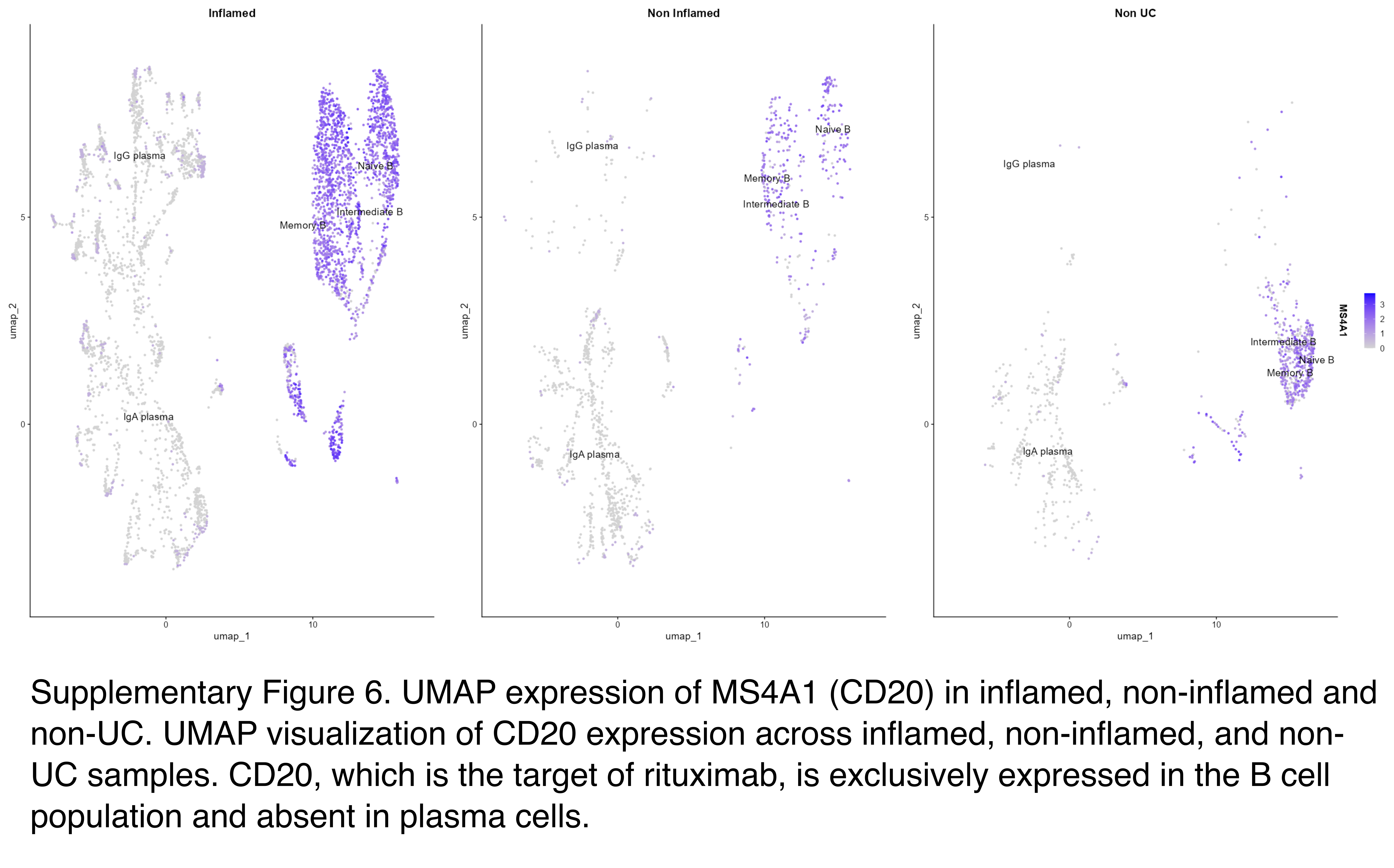

Supplement: izaf275_Supplementary_Data [file izaf275_supplementary_data.zip › Supplementary Figure 6.tiff]
